# Supplementary material for: Cloud BioLinux: pre-configured and on-demand bioinformatics computing for the genomics community
Source: BMC Bioinformatics. 2012 Mar 19;13:42. doi: 10.1186/1471-2105-13-42 (PMC3372431; doi:10.1186/1471-2105-13-42)
Supplement: Additional file 1 — Supplementary 1 Cloud BioLinux software documentation in the form of a mini, self-contained website. Users need to download and uncompress the .zip file, and open through a web browser the "index.html" file available on the main directory. (ZIP 1823 kb). [file 1471-2105-13-42-S1.ZIP › Cloud-BioLinux-Package-Documentation/docs/lfasta.html]

Bio-Linux Software Documentation Pages

Back to search form

## lfasta

|  |  |
| --- | --- |
| Name | lfasta |
| Description | **lfasta** is part of the fasta2 package. FASTA contains many programs for searching DNA and protein databases and for evaluating statistical significance from randomly shuffled sequences.  **lfasta** uses the fasta algorithm and should be chosen over **lalign** for aligning long sequences. For aligning peptides, see also **lalign** and **plalign**.  **References:**  Pearson, W.R. Flexible sequence similarity searching with the FASTA3 program package. Methods Mol Biol. 2000;132:185-219 [Entrez]    Pearson, W.R. Empirical statistical estimates for sequence similarity searches. J Mol Biol. 1998 Feb 13;276(1):71-84 [Entrez]    Pearson WR, Wood T, Zhang Z, Miller W. Comparison of DNA sequences with protein sequences. Genomics. 1997 Nov 15;46(1):24-36. [Entrez] |
| Homepage | http://www.people.virginia.edu/~wrp/pearson.html |
| Remote Documentation | http://www.people.virginia.edu/~wrp/papers/ismb2000.pdf |
